# The Impact of Polymorphic Variations in the 5p15, 6p12, 6p21 and 15q25 *Loci* on the Risk and Prognosis of Portuguese Patients with Non-Small Cell Lung Cancer

**DOI:** 10.1371/journal.pone.0072373

**Published:** 2013-09-06

**Authors:** Ramon Andrade de Mello, Mónica Ferreira, Filipa Soares-Pires, Sandra Costa, João Cunha, Pedro Oliveira, Venceslau Hespanhol, Rui Manuel Reis

**Affiliations:** 1 Department of Medicine, Faculty of Medicine, University of Porto (FMUP), Porto, Portugal; 2 Department of Medical Oncology, Instituto Português de Oncologia Francisco Gentil (IPO PORTO), Porto, Portugal; 3 Life and Health Sciences Research Institute (ICVS), School of Health Sciences, University of Minho, Braga, Portugal; 4 3B’s, PT Government Associate Laboratory, Braga/Guimarães, Portugal; 5 Department of Pneumology, Centro Hospitalar de São João, Faculty of Medicine, University of Porto, Porto, Portugal; 6 Department of Pneumology, Hospital São Marcos, Braga, Portugal; 7 Department of Populations Studies, Abel Salazar Biomedical Sciences Institute – ICBAS, University of Porto, Porto, Portugal; 8 Molecular Oncology Research Center, Barretos Cancer Hospital, Barretos-SP, Brazil; Nanjing Medical University, China

## Abstract

**Introduction:**

Polymorphic variants in the 5p15, 6p12, 6p21, and 15q25 *loci* were demonstrated to potentially contribute to lung cancer carcinogenesis. Therefore, this study was performed to assess the role of those variants in non-small cell lung cancer (NSCLC) risk and prognosis in a Portuguese population.

**Materials and Methods:**

Blood from patients with NSCLC was prospectively collected. To perform an association study, DNA from these patients and healthy controls were genotyped for a panel of 19 SNPs using a Sequenom® MassARRAY platform. Kaplan-Meier curves were used to assess the overall survival (OS) and progression-free survival (PFS).

**Results:**

One hundred and forty-four patients with NSCLC were successfully consecutively genotyped for the 19 SNPs. One SNP was associated with NSCLC risk: rs9295740 G/A. Two SNPs were associated with non-squamous histology: rs3024994 (VEGF intron 2) T/C and rs401681 C/T. Three SNPs were associated with response rate: rs3025035 (VEGF intron 7) C/T, rs833061 (VEGF –460) C/T and rs9295740 G/A. One SNP demonstrated an influence on PFS: rs401681 C/T at 5p15, p = 0.021. Four SNPs demonstrated an influence on OS: rs2010963 (*VEGF* +405 G/C), p = 0.042; rs3025010 (*VEGF* intron 5 C/T), p = 0.047; rs401681 C/T at 5p15, p = 0.046; and rs31489 C/A at 5p15, p = 0.029.

**Conclusions:**

Our study suggests that SNPs in the 6p12, 6p21, and 5p15 *loci* may serve as risk, predictive and prognostic NSCLC biomarkers. In the future, SNPs identified in the genomes of patients may improve NSCLC screening strategies and therapeutic management as well.

## Introduction

Lung cancer is an aggressive disease that affects more than 1.5 million people worldwide [1]. An American study estimated that there were 226,160 lung cancer cases and 160,340 lung cancer-related deaths for both sexes [2], corresponding to 29 and 14% of all cancer-related deaths in men and women, respectively [2]. In 2008, approximately 3,000 new lung cancer cases and approximately the same number of lung cancer-related deaths were reported in Portugal [3], [4]. Non-small cell lung cancer (NSCLC) represent 85% of lung cancer cases [5]. Recently, a randomized trial demonstrated the superiority of lung cancer screening using low-dose computed tomography compared with standard X-rays and presented a 20% reduction in death [6]. Thus, optimizing screening tools in a high-risk population is important for clinicians attempting to reduce lung cancer incidence and mortality.

Many risk factors are known to be responsible for lung cancer susceptibility, including tobacco consumption [7], passive smoke and occupational diseases [8], [9]. Moreover, the genomic profile has recently emerged as a major contributor to lung cancer carcinogenesis [10]–[23]. In 2009, a genome-wide associated study (GWAS) reported that a polymorphic variant in the 5p15.33 *locus*, rs2736100 (*TERT*), was associated with lung adenocarcinoma susceptibility [10]. In 2012, Ito *et al.* demonstrated that variants located in the CHRNA5-CHRNA3-CHRNB4 cluster on chromosome 15q25 (rs12914385, rs1317286, and rs931794) modified the impact of cigarette smoking on lung cancer risk in a Japanese population but demonstrated no statistically significant primary effects on lung cancer risk [11]. Our group demonstrated the impact of epidermal growth factor +61 A/G polymorphisms (located on chromosome 4q25–q27) on NSCLC risk in a Portuguese population [13].

Angiogenesis is known to play a major role in NSCLC carcinogenesis [5]. Vascular endothelial growth factor (VEGF) and its receptor (VEGFR) are considered the main catalysts of new vessel creation and tumor angiogenesis [24]. A recent systematic review reported that many polymorphic variations in the 6p12 and 6p21 *loci* could contribute to serum VEGF expression modulation and may thus influence tumor risk and prognosis [5]. Importantly, current anti-angiogenic therapies[4], [25]–[27], are in clinical use, and the genetic make-up of patients, particularly the allelic variants of the angiogenic proteins (e.g., VEGF and VEGFR), may modulate the patient response to those therapies. Thus, considering all of the paramount features of the polymorphic variations in the 5p15, 6p12, 6p21 and 15q25 *loci* and NSCLC tumor behavior, our group conducted this study to assess the role of the genetic polymorphisms in the 5p15, 6p12, 6p21 and 15q25 *loci* on NSCLC risk, with a secondary aim of assessing the role of those variants in patient outcome.

## Materials and Methods

### Design and Setting

A case-controlled/prospective study from February 2010 to April 2011 was conducted in two central North Portugal Hospitals: São João Hospital University Center, Porto, Portugal and São Marcos Hospital, Braga, Portugal. Laboratory studies were centralized at the Life and Health Sciences Research Institute, School of Health Sciences, University of Minho, Braga, Portugal.

### Subjects

For the statistical preparation of this study, sample calculations were performed prior to patient recruitment using the 95% confidential interval (CI) formula, and we considered a genotype proportion difference among cases and controls ranging from 10 to 20%. We used Piface software(http://homepage.stat.uiowa.edu/~rlenth/Power/) and estimated the sample number for approximately 126 patients for a power >0.8 and a *p*<0.05.

The inclusion criteria for this study included patients with a confirmed NSCLC diagnosis by histopathological examination, more than 21 years old at admission, a recommendation for treatment at participant’s institution, and informed consent after explanation of the study’s features by one of the researchers.

All of the patient follow-up information was obtained by consulting clinical records. The lung cancer patient median follow-up was 12 months. For NSCLC risk assessment, a group of blood donor controls were selected from a control set to match for gender and adjust for age in the statistical analysis. The control group had no follow-up information. Signed informed consent was obtained from each participant. The São João University Hospital Regional ethics committee approved this study. All patients and controls involved in this study were Portuguese Caucasians.

### First-line Treatment Data

Patients with advanced NSCLC with epidermal growth factor receptor (*EGFR)* exon 19 and 21 mutations orally received 250 mg of gefitinib once a day until disease progression was observed. *EGFR*-negative patients with advanced NSCLC were treated with a platinum-based regimen, according to clinical condition and indications.

### Variable Considered

The following demographic and clinical data were collected: age at admission, sex, ethnicity, histological diagnosis, TNM clinical stage (according to the American joint committee on cancer (AJCC) 2010 guidelines) [28], [29], Eastern Cooperative Oncology Group (ECOG) performance status score at admission [30], smoking status, *EGFR* mutation status, pack-years of cigarette consumption, overall survival (OS), and progression-free survival (PFS). The systemic therapy response rate (RR) was assessed by radiologists at our institution, according to (Response Evaluate Criteria in Solid Tumor) RECIST guidelines version 1.1 [31].

### Genotyping

DNA was extracted from the leukocytes of blood samples using the commercial Citogene® Blood Kit according to the manufacturer’s recommendations. [32] The extracted genomic DNA was analyzed by agarose gel electrophoresis, quantified by nanodrop, and stored at –70°C until use. The *loci* examined in this study are summarized in Table 1. Genotyping of the allele-specific primer extension products, which were generated from amplified DNA sequences, was performed using the Sequenom MassARRAY iPLEX Gold platform (Sequenom, San Diego, California) at the Instituto Gulbenkian de Ciências, Lisbon, Portugal. Primers were designed using MassARRAY Assay Design 3.1 software (Sequenom, San Diego, California) and genotyping was performed by an investigator who was blinded to the sample status (i.e., from case or control subjects). The genotyping quality was assessed by duplicate analysis of 10% of the samples, which demonstrated a 100% agreement rate.

**Table 1:** Single nucleotide polymorphisms primers sequence of the study.

Locus	Gene	SNP	Reverse	Forward
**5p15**	**TERT, CLPTM1L C/T**	**rs4635969**	ACGTTGGATGGAGATTAATGACAGGCCAAG	ACGTTGGATGGCATTTTTTTTCCTTTTGG
	**CLPTM1L C/A**	**rs31489**	ACGTTGGATGACAGCGAGACCTTGTCTCAA	ACGTTGGATGCTCGCATTCCACCTGTTTAC
	**CLPTM1L C/T**	**rs401681**	ACGTTGGATGAGGTCTGCTATCCAGACAAC	ACGTTGGATGGCTCTCCAAAGTTGTCGTAG
	**CLPTM1L C/T**	**rs402710**	ACGTTGGATGTCTACCTGTACCAGCGGTG	ACGTTGGATGCGGTGAAAGCCGTCATTCC
**6p12**	**VEGF intron 7 C/T**	**rs3025035**	ACGTTGGATGGGTTTGTGTGAAGTGACCTG	ACGTTGGATGTATTCCCAGATACAGCCAGC
	**VEGF +936 C/T**	**rs3025039**	ACGTTGGATGAGCACTTTGGGTCCGGAGG	ACGTTGGATGATGGCGAATCCAATTCCAAG
	**VEGF 3′-UTR T/C**	**rs3025040**	ACGTTGGATGATCCCCAAAGCACAGCAATG	ACGTTGGATGAGATCACAGGTACAGGGATG
	**VEGF -2489 C/T**	**rs1005230**	ACGTTGGATGTCAGAGCCCCAACTTTGTTC	ACGTTGGATGGCATATAGGAAGCAGCTTGG
	**VEGFA - 2578 C/A**	**rs699947**	ACGTTGGATGGTCAGTCTGATTATCCACC	ACGTTGGATGTTCCCATTCTCAGTCCATGC
	**VEGF –460 C/T**	**rs833061**	ACGTTGGATGTTGGAATCCTGGAGTGACCC	ACGTTGGATGTGTGGGTGAGTGAGTGTGTG
	**VEGF intron 2 A/G**	**rs833070**	ACGTTGGATGAAGTTCACAGCACCCGAACA	ACGTTGGATGCCCTGGTTTGCATTCCTTTG
	**VEGF intron 5 C/T**	**rs3025010**	ACGTTGGATGCCCTTCAAGAGAACCAGAGC	ACGTTGGATGCTTTCTTCCCTGTGACAGAC
	**VEGF intron 2 T/C**	**rs3024994**	ACGTTGGATGATGGGCACAGAATCCTTCTC	ACGTTGGATGTCAGACTTCTAGTCTCGTTC
	**VEGF –7 C/T**	**rs25648**	ACGTTGGATGCACAGCCCGAGCCGGAGAG	ACGTTGGATGGCACCCAAGACAGCAGAAAG
	**VEGF +405 G/C**	**rs2010963**	ACGTTGGATGCGGCGGTCACCCCCAAAAG	ACGTTGGATGTCGAGGAAGAGAGAGACGG
**6p21**	**vWF G/A**	**rs9295740**	ACGTTGGATGCACACAGCTCAATATGGTCC	ACGTTGGATGGTGGACAGAAAGGCAGTTCA
**15q25**	**CHRNA3 C/T**	**rs12914385**	ACGTTGGATGGCAAAAAAACAGAAGATGTC	ACGTTGGATGGGCTCTATTTTTGTAGTTGC
	**LOC123688 T/C**	**rs8034191**	ACGTTGGATGCCACAAGTCCCCTTAGTTAC	ACGTTGGATGGTAGTGGTTAGAGCCCAATG
	**AGPHD1 G/A**	**rs931794**	ACGTTGGATGGCTTGCTTGTGGTACTTTTG	ACGTTGGATGGACAGAGCACATGAAATCCC

**Abbreviations**: SNP, single nucleotide polymorphisms; vWF, von Willebrand factor; VEGF, vascular endothelial growth factor; CHRNA3, cholinergic nicotine receptor alpha3; VEGF 3′-UTR, VEGF 3′untranslated region; TERT, telomerase reverse transcriptase; CLPTM1L, cleft lip and palate transmembrane 1-like; AGPHD1, aminoglycoside phosphotransferase domain containing 1.

### Statistical Analysis

*Χ^2^* and Wilcoxon-Mann Whitney tests were used to compare the frequency distribution of the age, sex, and genetic polymorphisms at the 5p15, 6p12, 6p21 and 15q25 *loci* and the allele distribution among the cases and controls. Moreover, the *Χ^2^* test was used to verify that the observed allele distribution in the control group was in Hardy-Weinberg equilibrium (HWE). The odds ratio (OR) and 95% CI for the effect of the polymorphic variants on the risk for NSCLC were estimated using univariate and multivariate logistic regression analyses, which were adjusted for sex and age as continuous variables. The false-positive report probability (FPRP) was calculated for significant associations observed in multivariate tests according to the study by Wacholder and colleagues [33]. Furthermore, we analyzed OS and PFS using Kaplan-Meier curves. All statistical tests were two-sided, and significance was considered for *p*<0.05. Data analysis was performed using IBM® SPSS Statistics, version 19.0.

## Results

### Patient’s Clinical-pathological Data

During the study period, we consecutively enrolled 144 patients with NSCLC and 144 controls (Table 2). The median age was 61 years (range: 32–89) in the NSCLC group and 48 years (range: 35–65) in the control group. Tables 3 and 1S demonstrate the relationship between the distribution of the polymorphic variant allele frequencies in the control group and Hardy-Weinberg equilibrium. All controls were in HWE. The sex distribution proportions were the same among the patients with NSCLC and controls (matched 1∶1).

**Table 2:** Summary of clinical-pathological features of subjects and controls.

Characteristics	Cases	Controls
**Number**	144	144
**Gender**		
** Male**	78.5%	78.5%
** Female**	21.5%	21.5%
Age*	61.5 (32–89)	48 (35–65)
** <41 years**	2.8%	16%
** >40 years**	97.2%	84%
**Smoke status**		
** Current smoke**	45.3%	n.a
** Ex - smoke**	37.9%	n.a
** Never - smoke**	16.8%	n.a
Packs-year*	40 (0–240)	n.a
**Histology**		
** Non-squamous**	77.1%	–
** Squamous Cell**	22.9%	–
***EGFR*** ** mutation**		
** Positive**	12.2%	n.a.
** Negative**	87.8%	n.a.
**TNM stage**		–
** I–IIIA**	8.6%	–
** IIIB**	12.4%	–
** IV**	79%	–
**ECOG PS**		
** 0–2**	88.6%	100%
** 3–4**	11.4%	0.00
PFS (months) *	5 (3.95–6.40)	n.a.
OS (months) *	10 (8.11–11.88)	n.a.

**Abbreviations**: *median; EGFR – epidermal growth factor receptor; ECOG PS, eastern cooperative oncology group performance status scale; n.a - not assessed; OS, overall survival; PFS, progression-free-survival.

**Table 3:** Genotypes of the single nucleotide polymorphisms at 5p15, 6p12, 6p21, and 15q25 *loci* in lung cancer patients and control subjects and their association with the risk of non-small-cell lung cancer adjusted for age and gender.

Chromosome region	Genotype				Control n (%)	NSCLC n (%)	NSCLC (OR, 95%CI)	HWE (*p* value)
**6p21**	**rs9295740 (vWF G/A)**							
	GG				94 (65.3)	84 (58.3)	–	
	GA				42 (29.2)	54 (37.5)	**1.978 (1.076–3.636)**	
	AA				8 (5.6)	6 (4.2)	0.814 (0.229–2.895)	0.263
	GA+AA vs GG				50 (34.8)	60 (41.7)	**1.742 (0.979–3.098)**	
	Allele			G	79.86	77.08		
	A				20.14	22.92		
**15q25**	**rs12914385 (CHRNA3 C/T)**							
	CC				51 (35.4)	39 (27.1)	–	
	CT				69 (47.9)	72 (50.0)	**1.835 (0.964–3.490)**	
	TT				24 (16.7)	33 (22.9)	1.844 (0.824–4.126)	0.935
	CT+TT vs CC				93 (64.6)	105 (72.9)	**1.837 (1.002–3.369)**	
	Allele		C		59.38	58.82		
	T				40.62	41.18		
**15q25**	**rs8034191 (LOC123688 T/C)**							
	TT				53 (36.8)	44 (30.6)	–	
	TC				67 (46.5)	71 (49.3)	**1.785 (0.947–3.362)**	
	CC				24 (16.7)	29 (20.1)	1.424 (0.635–3.195)	0.718
	TC+CC vs TT				91 (63.2)	100 (69.4)	**1.674 (0.925–3.032)**	
	Allele	T			60.07	61.39		
	C				39.93	38.61		

**Abbreviations:** NSCLC, non-small-cell lung cancer; vWF, von Willebrand factor; CHRNA3, cholinergic nicotine receptor alpha3; HWE, Hardy-Weinberg equilibrium. In HWE column p>0.05 stands for control group in HWE. **Bold** was used for highlight the almost statistical significance results and **bold+gray** for statistic significant results.

### Susceptibility Assessment

By univariate and multivariate analyses adjusted for gender and age (Tables 3 and 1S), we found a significant association between rs9295740 G/A (6p21) and the overall NSCLC risk (OR = 1.978, 95% CI: 1.076–3.636), which was mainly in males (OR = 1.921, 95% CI: 0.942–3.919). We observed that the rs9295740 GA+AA genotype group represented a nearly statistically significant increase in NSCLC risk when compared with the rs9295740 AA genotype group (OR = 1.742, 95% CI: 0.979–3.098). Furthermore, we found a nearly statistically significant increase in the overall NSCLC risk for the rs12914385 (*CHRNA3*) C/T polymorphic variation (OR = 1.835, 95% CI: 0.964–3.490) when compared with the rs12914385 (*CHRNA3*) C/C variant(Table 3). Moreover, the rs12914385 (*CHRNA3*) CT+TT genotype group demonstrated a significant association with NSCLC risk (OR = 1.837, 95% CI: 1.002–3.369).

When assessing the association between the polymorphic variants in the 5p15, 6p12, 6p21 and 15q25 *loci* and histological subtype, we noted an association between the two genetic polymorphisms rs3024994 (*VEGF* intron 2) T/C and rs401681 (CLPTM1L) C/T and the non-squamous histological subtype (OR = 5.315, 95% CI: 1.256–22.479 and OR = 3.273, 95% CI: 1.006–10.648, respectively).

A FPRP calculation demonstrated that all of the above mentioned significant polymorphism associations (i.e., rs9295740 (vWF G/A), rs12914385 (CHRNA3 C/T), rs3024994 (VEGF intron 2 T/C), and rs401681 (CLPTM1L C/T)) remained significant (FPRP≤0.5) when a prior association probability ≥10% was considered.

### Predictive Biomarkers (First Line Regimen Response Rate)

Table 4 summarizes the relationships among the polymorphic variations studied herein and NSCLC outcome mainly with respect to the RR of the first-line regimen, PFS and OS. For the 5p15 *locus*, we found that the rs4635969 (*TERT*) CT+TT genotype group had a 44.4% overall RR when compared with the rs4635969 CC genotype, *p* = 0.006(Table 4). We found that three polymorphic variations in the 6p12 *locus* were predictive biomarkers for the overall platinum based regimen. The rs3025035 (*VEGF* intron 7) CT genotype had a higher overall RR than the rs3025035 (*VEGF* intron 7) CC genotype (72.2% versus 35.5%, *p* = 0.005). The rs833061 (*VEGF* –460) CT genotype had a slightly lower RR than the rs833061 (*VEGF* –460) CC genotype (42.9% versus 43.8%, *p* = 0.036). The rs833070 (*VEGF* intron 2) AG+GG genotype group had a little inner RR than the AA genotype group (42.6% versus 43.8%, *p* = 0.011). The rs9295740 G/A genotype in the 6p21 *locus* had a higher overall RR than the rs9295740 GG genotype (50% versus 41.8%, *p* = 0.042; Table 4). We found no predictive biomarkers for the first-line platinum based regimen in the 15q25 *locus*.

**Table 4:** Genetic polymorphisms and non-small-cell lung cancer outcome (PFS, OS, RR for 1th line regimen).

Chromosome region	Genotype	PFS (months)*	*P* value	OS (months)*	*P* value	Positive RR (%)	*P* value	Positive RR (OR, 95% CI)^†^
**5p15**	**rs4635969 C/T (TERT)**							
	CC	5 (3.38–6.61)		9 (6.39–11.60)		41.4		–
	CT	5 (1.94–8.05)	0.665 A	15 (9.17–20.83)	0.337 A	41.9	0.66^C^	1.036 (0.427–2.517)
	TT	6 (0.001–16.73)		11 (0.265–21.73)		60.01		1.918 (0.290–12.672)
	CT+TT vs CC	5 (2.21–7.78)	0.445 A	13 (8.82–17.17)	0.417 A	**44.4**	**0.006** C	1.129 (0.486–2.623)
	**rs31489 (CLPTM1L C/A)**							
	CC	4 (1.89–6.10)		**6 (1.104–10.896)**		33.3		–
	CA	6 (4.16–7.83)	0.588^A^	**13 (6.33–19.66)**	**0.029** F	43.5	0.316 C	0.408 (0.122–1.360)
	AA	6 (1.42–10.57)		**13 (6.98–19.01)**		55.6		0.625 (0.208–1.879)
	CA+AA vs CC	6 (4.42–7.57)	0.449 A	**13 (7.89–18.1)**	**0.008** F	46.9	0.216 C	0.571 (0.231–1.416)
	**rs401681 (CLPTM1L C/T)**							
	CC	**2 (0.945–3.05)**		**6 (3.02–8.97)**		33.3		
	CT	**5 (3.22–6.77)**	**0.021** D	**13 (8.61–17.38)**	**0.046** A	43.1	0.442 C	1.488 (0.538–4.116)
	TT	**7 (0.001–14.04)**		**10 (5.10–14.89)**		52.6		2.149 (0.620–7.455)
	CT+TT vs CC	6 (4.5–7.49)	0.332^A^	**11 (8.38–13.61)**	**0.021** A	80	0.29 C	1.644 (0.620–4.359)
**6p12**	**rs3025035 (VEGF intron 7 C/T)**							
	CC	5 (3.55–6.44)		10 (7.28–12.71)		35.5		–
	CT	8 (3.88–12.11)	0.282 A	12 (4.35–19.65)	0.472 A	**72.2**	**0.005** C	**4.90 (1.56–15.37)**
	TT	–		–		–		–
	**rs833061 (VEGF –460 C/T)**							
	CC	3 (0.0001–6.76)		9 (5.38–12.61)		**43.8**		–
	CT	5 (3.82–6.18)	0.752 A	11 (8.47–13.52)	0.975 A	**42.9**	**0.036** C	1.028 (0.331–3.196)
	TT	5 (0.939–9.061)		10 (3.06–16.92)		**40.9**		0.967 (0.258–3.626)
	CT+TT vs CC	5 (3.84–6.15)	0.469 A	10 (8.14–11.86)	0.902 A	**42.3**	**0.011** C	1.011 (0.336–3.039)
	**rs833070 (VEGF intron 2 A/G)**							
	AA	3 (0.82–5.18)		9 (2.09–15.90)		43.8		–
	AG	6 (4.68–7.31)	0.424^A^	11 (8.05–13.94)	0.569 B	46	0.788^C^	1.172 (0.372–3.697)
	GG	5 (2.64–7.35)		9 (3.77–14.22)		35.7		0.767 (0.216–2.728)
	AG+GG vs AA	5 (3.83–6.17)	0.206^A^	10 (7.41–12.58)	0.471^B^	**42.6**	**0.011** C	1.011 (0.336–3.039)
	**rs3025010 (VEGF intron 5 C/T)**							
	CC	4 (1.85–6.14)		**6 (3.16–8.83)**		38.1		
	CT	6 (4.35–7.64)	0.977^A^	**13 (9.63–16.36)**	**0.047** D	41.5	0.307 C	1.163 (0.481–2.81)
	TT	6 (1.01–10.98)		**10 (0.0001–20.22)**		63.6		2.733 (0.685–10.90)
	CT+TT vs CC	6 (4.39–7.60)	0.883 A	**13 (8.22–11.17)**	**0.020** F	46.2	0.530 C	1.393 (0.979–1.053)
	**rs2010963 (VEGF +405 G/C)**							
	GG	5 (3.1–6.89)		**9 (4.17–13.82)**		47.1		–
	GC	5 (3.12–6.87)	0.463^A^	**13 (8.70–17.29)**	**0.042** G	43.2	0.570 C	0.876 (0.354–2.164)
	CC	5 (1.67–8.32)		**3 (0.0001–8.88)**		31.3		0.503 (0.143–1.771)
	GC+CC vs GG	5 (2.98–7.01)	0.459 A	10 (5.56–14.43)	0.441 G	40	0.506 C	0.759 (0.324–1.779)
**6p21**	**rs9295740 (vWF G/A)**							
	GG	**4 (2.06–5.93)**		9 (5.91–12.08)		**41.8**		–
	GA	**6 (3.21–8.78)**	**0.074** A	13 (8.4–17.59)	0.107 A	**50**	**0.042** C	1.385 (0.584–3.285)
	AA	**4 (0.39–7.60)**		11 (8.6–13.4)		0.0001		–
	GA+AA vs GG	**6 (4.31–7.68)**	**0.034** A	**13 (9.38–16.61)**	**0.045** A	43.6	0.864 C	1.066 (0.464–2.452)

**Abbreviations**: * median; PFS, progression-free-survival; OS, overall survival; RR, response rate for 1th line therapy (complete, partial, stable); 95% CI, confidential interval 95%.

A Log rank test;

B Breslow test;

C Chi square test;

D Breslow test (only non-squamous cell tumors);

E Log rank test (only stages IIIB and IV);

F Log rank test (only non-squamous cell tumors);

G Breslow test (only stages IIIB and IV). **Bold** was used for highlight almost statistical significance results and **bold+gray** for statistic significant results.; ^†^adjusted for age**.**

### Progression-free Survival

**Figure 1:**
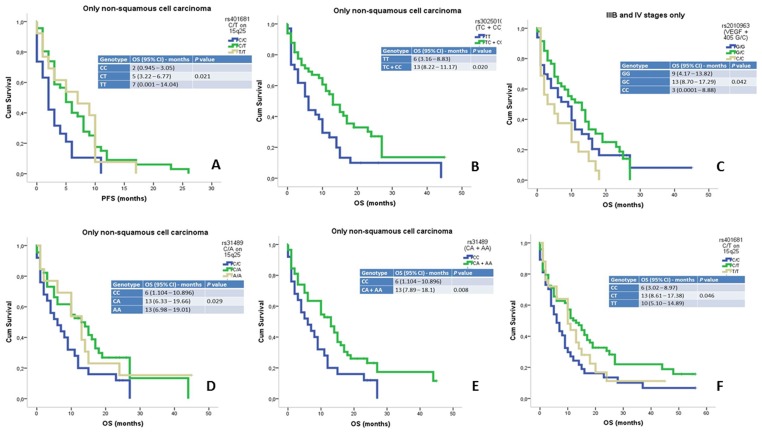
Progression-free survival (PFS) curve for the rs401681 C/T genetic polymorphism (A). Kaplan-Meir curve of the overall survival (OS) for the rs3025010 (*VEGF* intron 5) C/T genetic polymorphisms (B). Kaplan-Meir curve of the overall survival (OS) for the rs2010963 (*VEGF* +405) G/C genetic polymorphisms (C). Kaplan-Meir curve of the overall survival (OS) for the rs31489 C/A genetic polymorphisms (D). Kaplan-Meir curve of the overall survival (OS) for the rs31489 CA+AA genotypes versus the rs31489 CC genotype (E). Kaplan-Meir curve of the overall survival (OS) for rs401681 C/T genetic polymorphisms (F).

The overall PFS of the cohort was 5 months (range: 3.95–6.40). The PFS and genetic polymorphism data are summarized in Tables 4 and 2S. Only two *loci* had polymorphic variants with an impact on PFS: rs9295740 G/A (6p21) and rs401681 C/T (15q25). The rs9295740 GA genotype in the 6p21 *locus* demonstrated a trend toward higher PFS than the rs9295740 GG and rs9295740 AA genotypes: 6 months (range: 3.21–8.78) versus 4 months (range: 2.06–5.93) versus 4 months (range: 0.39–7.60), respectively, *p* = 0.074. However, the rs9295740 GA+AA genotype group had a higher PFS than the rs9295740 GG genotype group: 6 months (range: 4.31–7.68) versus 4 months (range: 2.06–5.93), respectively, *p* = 0.034. The 5p15 *locus* rs401681 TT genotype had a higher PFS than the rs401681 CC and rs401681 CT genotypes: 7 months (0.001–14.04) versus 2 months (range: 0.945–3.05) versus 5 months (range: 3.22–6.77) months, respectively, *p* = 0.021(Figure 1A).

### Overall Survival

The OS of the study population was 10 months (range: 8.11–11.88). The polymorphic variants in the 6p12, 6p21 and 5p15 *loci* were significantly associated with OS (Table 4). Two polymorphic variants in the 6p12 *locus* were associated with OS. In only the non-squamous histological subtype, the rs3025010 (*VEGF* intron 5) CT genotype had a higher OS than the rs3025010 (VEGF intron 5) TT and rs3025010 (VEGF intron 5) CC genotypes: 13 months (range: 9.63–16.36) versus 10 months (range: 0.0001–20.22) versus 6 months (range: 3.16–8.83), respectively, *p* = 0.047(Figure 1B). Furthermore, in only stages IIIB and IV, the rs2010963 (*VEGF* +405) GC genotype had a higher OS than the rs2010963 (*VEGF* +405) GG and rs2010963 (*VEGF* +405) CC genotypes: 13 months (range: 8.70–17.29) versus 9 months (range: 4.17–13.82) versus 3 months (range: 0.0001–8.88), respectively, *p* = 0.042(Figure 1C). The rs9295740 GA+AA genotype group in the 6p21 *locus* had a higher OS than the rs9295740 GG genotype group: 13 months (range: 9.38–16.61) versus 9 months (range: 5.91–12.08), *p* = 0.045.

Two polymorphic variants were associated with OS in the 5p15 *locus*. We observed that in only the non-small histology subtype, the rs31489 AA and rs31489 CA genotypes had a higher OS than the rs31489 CC genotype: 13 months (range: 6.98–19.01) versus 13 months (range: 6.33–19.66) versus 6 months (range: 1.104–10.896), respectively, *p* = 0.029(Figure 1D and 1E). Moreover, the rs401681 CT genotype had a higher OS than the rs401681 TT and rs401681 CC genotypes: 13 months (range: 8.61–17.38) versus 10 months (range: 5.10–14.89) versus 6 months (range: 3.02–8.97), respectively, *p* = 0.046(Figure 1F).

## Discussion

### NSCLC Risk Biomarkers

In 2009, Landi *et al.* reported that nicotinic acetylcholine receptor gene variants on chromosome 15q25 were associated with an elevated overall lung cancer risk [10]. These single nucleotide polymorphisms (SNPs) were also strongly associated with all major histological groups of patients who were current and former smokers [10]. In our study, the rs9295740 G/A genotype (6p21) was associated with NSCLC risk in Portugal mainly in males. In 2012, Bae *et al.* reported results from a Korean study that assessed 1,094 Korean patients and 1,100 healthy controls [12]. The authors did not find any association between rs9295740 G/A polymorphisms and lung cancer risk in a Korean population. This fact may be explained by divergences in the genomic expression of different populations, as previously reported [34]. Furthermore, we found a trend for association with risk assessment and conditions, including NSCLC risk in males and rs833061 (*VEGF* –460) C/T polymorphisms, overall NSCLC and rs12914385 *CHRNA3* C/T polymorphisms, overall NSCLC and rs8034191 (LOC123688) T/C polymorphisms and NSCLC risk in males and rs931794 G/A polymorphisms. Previous Asiatic studies [11], [12] also reported polymorphic variants in the 15q25 *locus* and NSCLC risk, suggesting that instabilities in the 15q25 *locus* could be a major mediator of lung cancer carcinogenesis. Though the 5p15 *locus* was previously reported [35] to be associated with lung cancer susceptibility due to telomerase reverse transcriptase and cleft lip and palate transmembrane 1-like gene effects, we could not find an association between rs4635969 C/T polymorphisms and NSCLC risk. In the 6p12 *locus*, we observed a nearly statistically significant association between the rs833061 (VEGF –460 C/T) polymorphism and NSCLC risk in males. In 2008, Zhai *et al.* conducted a study that assessed 1,900 cases and 1,458 controls in a Caucasian population [17]. Zhai and colleagues found no significant association between rs833061 and NSCLC, suggesting that the 6p12 *locus* has no relationship with overall lung cancer susceptibility. Nevertheless, our study demonstrated that variants in the 6p12 and 15q25 *loci* such as rs3024994 (*VEGF* intron 2) T/C and rs401681 C/T, respectively, were significantly associated with non-squamous lung cancer histology risk. In 2012, a Chinese group assessed 196 lung cancer patients and 229 healthy controls [36]. This group found that multiple 5p15 variants contributed to lung adenocarcinoma susceptibility [36].

### NSCLC Predictive Biomarkers

*EGFR* mutations in exons 19 and 21 are the most effective, predictive biomarkers of the response to EGFR TKIs for first-line advanced NSCLC treatment [37], [38]. In this context, finding novel predictive biomarkers for overall systemic treatment remains a challenge for clinicians and researchers. Moreover, three polymorphic variants in the 6p12 and 6p21 *loci* demonstrated a significant influence on the overall response rate of first-line systemic therapies regardless of the chosen regimen including rs3025035 (*VEGF* intron 7) C/T, rs833061 (*VEGF* –460) C/T, and rs9295740 G/A. These results may potentially lead to useful biomarkers for therapeutic prediction. Importantly, these genetic polymorphisms may be assessed using blood samples, increasing their clinical application. Meanwhile, these findings may support the idea that VEGF modulation may be a key player in lung cancer carcinogenesis and aggressiveness, which was previously suggested in pre-clinical and translational models [5], [39].

### NSCLC Prognostic Biomarkers

Our study also evaluated NSCLC prognosis by assessing the 19 SNPs and their relationship with PFS and OS. Our studied demonstrated that a polymorphic variant in the 5p15 *locus*, rs401681 C/T, was associated with the PFS of non-squamous cell tumors. Previous reports [8] demonstrated that the histology subtype of patients with NSCLC is associated with different clinical behaviors among patients with NSCLC. We also found that another polymorphic variant in 6p21, rs9295740 G/A, demonstrated a nearly statistically significant influence on the PFS for all patients with NSCLC. We found that four SNPs were associated with the NSCLC overall survival (e.g., rs3025010 (*VEGF* intron 5) C/T (at 6p12), rs2010963 (*VEGF* +405) G/C (at 6p12), rs31489 C/A (at 5p15) and rs401681 C/T (at 5p15)). In 2008, Heist *et al.* [16] demonstrated that the rs2010963 (*VEGF* +405) GC genotype was associated with better survival than the GG and CC genotypes, which is in agreement with our results. However, in 2010, Dong *et al.* [20] screened 54 SNPs in 568 Chinese patients with NSCLC and assessed the association of the *VEGF* and *EGFR* genetic polymorphisms with NSCLC prognosis. This study did not find an association between rs3025010 (*VEGF* intron 5) C/T variants and NSCLC survival. GWAS [10], [11] and other studies [12], [36], [40] found that the 5p15 *locus* was associated with lung cancer susceptibility, but none of those studies reported concerns regarding overall survival. This is the first study to report that polymorphic variants e.g., rs31489 C/A and rs401681 C/T, in the 5p15 *locus* are associated with NSCLC overall survival and patient prognosis in a Portuguese population.

## Conclusions

Lung cancer management remains a challenge for researchers and clinicians worldwide. The comprehensive understanding of lung cancer biology is urgently needed. We believe that the results presented in this study provide additional findings for NSCLC understanding. We found that 1 SNP in the 6p21 *locus* is associated with NSCLC risk, 1 SNP in the 6p12 locus and 1 SNP in the 15q25 locus is associated with non-squamous histology, 1 SNP at 5p15 is associated with PFS, 2 SNPs at 5p15 and 2 SNPs at 6p12 are associated with OS, 2 SNPs at 6p12 and 1 SNP at 6p21 are associated with RR for first-line therapy. Our work suggests that variants on chromosomes 5p15 and 6p21 are prognostic biomarkers for advanced NSCLC. In addition, variants at 6p21 are NSCLC risk biomarkers, and variants at 6p12 and 6p21 are predictive NSCLC biomarkers. In the future, SNPs identified in the genomes of patients may improve NSCLC screening strategies and therapeutic management.

## Supporting Information

Table S1 **Case-control analysis of all 19 SNPs.**
(DOC)

Table S2 **Analysis of all 19 SNPs and patients outcome.**
(DOC)
