# Supplementary material for: The Impact of Polymorphic Variations in the 5p15, 6p12, 6p21 and 15q25 Loci on the Risk and Prognosis of Portuguese Patients with Non-Small Cell Lung Cancer
Source: PLoS One. 2013 Sep 6;8(9):e72373. doi: 10.1371/journal.pone.0072373 (PMC3765163; doi:10.1371/journal.pone.0072373)
Supplement: Table S1 — Case-control analysis of all 19 SNPs. (DOC) [file pone.0072373.s001.doc]

**Table 1 Supplementary -** Genotypes of the single nucleotide polymorphisms at 5p15, 6p12, 6p21, and 15q25 *loci* in lung cancer patients and control subjects and their association with the risk of non-small-cell lung cancer adjusted for age and gender.

| **Chromosome region** | **Genotype** | **Control n (%)** | **NSCLC n (%)** | **NSCLC (OR, 95%CI)** | **HWE (*p* value)** |
| --- | --- | --- | --- | --- | --- |
| **5p15** | **rs4635969 C/T (TERT)** |  |  |  |  |
|  | CC | 93(64.6) | 94(65.3) | - |  |
|  | CT | 47(32.6) | 44 (30.6) | 0.794 (0.439 – 1.437) |  |
|  | TT | 4 (2.8) | 6 (4.2) | 0.714 (0.147 – 3.470) | 0.500 |
|  | CT + TT vs CC | 51 (50.5) | 50 (49.5) | 0.787 (0.442 – 1.401) |  |
|  | Allele C | 89.02 | 80.56 | - |  |
|  | T | 19.09 | 19.44 |  |  |
|  | **rs31489 (CLPTM1L C/A)** |  |  |  |  |
|  | CC | 49 (34.0) | 48 (33.3) | - |  |
|  | CA | 74 (51.4) | 71 (49.3) | 0.854 (0.462-1.576) |  |
|  | AA | 21 (14.6) | 25 (17.4) | 1.184 (0.514-2.730) | 0.413 |
|  | CA + AA vs CC | 95 (66.0) | 96 (66.7) | 0.924 (0.517-1.653) |  |
|  | Allele C | 59.72 | 57.99 |  |  |
|  | A | 40.28 | 42.01 |  |  |
|  | **rs401681 (CLPTM1L C/T)** |  |  |  |  |
|  | CC | 44 (30.6) | 40 (27.8) | - |  |
|  | CT | 75 (52.1) | 77 (53.5) | 1.042 (0.556-1.956) |  |
|  | TT | 25 (17.4) | 27 (18.7) | 1.062 (0.467-2.416) | 0.471 |
|  | CT + TT vs CC | 100 (69.5) | 104 (72.2) | 1.047 (0.575-1.907) |  |
|  | Allele C | 56.60 | 54.51 |  |  |
|  | T | 43.40 | 45.49 |  |  |
|  | **rs402710 (CLPTM1L C/T)** |  |  |  |  |
|  | CC | 15 (10.4) | 20 (13.9) | - |  |
|  | CT | 64 (44.4) | 63 (43.8) | 0.484 (0.194-1.206) |  |
|  | TT | 65 (45.1) | 61 (42.4) | 0.629 (0.255-1.554) | 0.897 |
|  | CT + TT vs CC | 129 (89.5) | 124 (86.2) | 0.554 (0.236-1.304) |  |
|  | Allele C | 32.64 | 35.76 |  |  |
|  | T | 67.36 | 64.24 |  |  |
| **6p12** | **rs3025035 (VEGF intron 7 C/T)** |  |  |  |  |
|  | CC | 118 (81.9) | 116 (80.6) | - |  |
|  | CT | 26 (18.1) | 27 (18.8) | 0.848 (0.413-1.743) |  |
|  | TT | 0 (0) | 1 (0.7) | - | 0.234 |
|  | CT + TT vs CC | 26 (18.1) | 28 (19.5) | 0.892 (0.427-1.864) |  |
|  | Allele C | 90.97 | 89.93 |  |  |
|  | T | 9.03 | 10.07 |  |  |
|  | **rs3025039 (VEGF +936 C/T)** |  |  |  |  |
|  | CC | 111 (77.1) | 115 (79.9) | - |  |
|  | CT | 30 (20.8) | 28 (19.4) | 0.810 (0.409-1.602) |  |
|  | TT | 3 (2.1) | 1 (0.7) | 0.978 (0.086-11.074) | 0.568 |
|  | CT + TT vs CC | 33 (22.9) | 29 (20.1) | 0.789 (0.388-1.604) |  |
|  | Allele C | 87.50 | 89.58 |  |  |
|  | T | 12.50 | 10.42 |  |  |
|  | **rs3025040 (VEGF 3'-UTR T/C)** |  |  |  |  |
|  | TT | 3 (2.1) | 1 (0.7) | - |  |
|  | TC | 29 (20.1) | 29 (20.1) | 0.860 (0.071-10.353) |  |
|  | CC | 112 (77.8) | 114 (79.2) | 1.012 (0.089-11.461) | 0.495 |
|  | TC + CC vs TT | 141 (97.9) | 143 (99.3) | 0.812 (0.062-10.588) |  |
|  | Allele C | 87.85 | 89.23 |  |  |
|  | T | 12.15 | 10.77 |  |  |
|  | **rs1005230 ( VEGF -2489 C/T)** |  |  |  |  |
|  | CC | 44 (30.6) | 43 (29.9) |  |  |
|  | CT | 73 (50.7) | 75 (52.1) | 1.227 (0.650 – 2.314) |  |
|  | TT | 27 (18.8) | 26 (18.1) | 0.996 (0.444 – 2.235) | 0.735 |
|  | CT +TT vs CC | 100 (69.44) | 101 (70.13) | 1.159 (0.635 – 2.114) |  |
|  | Allele C | 55.90 | 55.90 | - |  |
|  | T | 44.01 | 44.01 | - |  |
|  | **rs699947 (VEGFA - 2578 C/A)** |  |  |  |  |
|  | CC | 44 (30.6) | 43 (29.8) | - |  |
|  | CA | 73 (50.7) | 75 (52.1) | 1.227 (0.650-2.314) |  |
|  | AA | 27 (18.8) | 26 (18.1) | 0.996 (0.444-2.235) | 0.735 |
|  | CA + AA vs CC | 100 (69.4) | 101 (70.2) | 1.056 (0.460-2.424) |  |
|  | Allele C | 55.90 | 61.45 |  |  |
|  | A | 44.10 | 38.55 |  |  |
|  | **rs833061 (VEGF – 460 C/T)** |  |  |  |  |
|  | CC | 31 (21.5) | 28 (19.4) | - |  |
|  | CT | 72 (50.0) | 79 (54.9) | 1.355 (0.664-2.764) |  |
|  | TT | 41 (28.5) | 37 (25.7) | 0.969 (0.433-2.167) | 0.954 |
|  | CT + TT vs CC | 113 (78.5) | 116 (80.6) | 1.985 (0.248-15.920) |  |
|  | Allele C | 46.53 | 46.87 |  |  |
|  | T | 53.47 | 53.13 |  |  |
|  | **rs833070 (VEGF intron 2 A/G)** |  |  |  |  |
|  | AA | 27 (18.8) | 26 (18.1) | - |  |
|  | AG | 72 (50.0) | 75 (52.1) | 1.259 (0.599-2.654) |  |
|  | GG | 45 (31.3) | 43 (29.8) | 0.971 (0.434-2.173) | 0.849 |
|  | AG + GG vs AA | 117 (81.3) | 118 (81.9) | 0.696 (0.080-6.015) |  |
|  | Allele A | 43.75 | 51.84 |  |  |
|  | G | 56.25 | 48.16 |  |  |
|  | **rs3025010 (VEGF intron 5 C/T)** |  |  |  |  |
|  | CC | 61 (42.4) | 62 (43.1) | - |  |
|  | CT | 71 (49.3) | 67 (46.5) | 1.084 (0.607-1.936) |  |
|  | TT | 12 (8.3) | 15 (10.4) | 1.489 (0.548-4.045) | 0.167 |
|  | CT + TT vs CC | 83 (57.6) | 82 (56.9) | 1.148 (0.504-2.614) |  |
|  | Allele C | 67.01 | 66.32 |  |  |
|  | T | 32.99 | 33.68 |  |  |
|  | **rs3024994 (VEGF intron 2 T/C)** |  |  |  |  |
|  | TT | 1 (0.7) | 0 (0) | - |  |
|  | TC | 11 (7.6) | 17 (11.8) | - |  |
|  | CC | 132 (91.7) | 127 (88.2) | - | 0.172 |
|  | TC + CC vs TT | 143 (99.3) | 144 (100) | - |  |
|  | Allele T | 4.51 | 5.90 |  |  |
|  | C | 95.49 | 94.10 |  |  |
|  | **rs25648 (VEGF – 7 C/T)** |  |  |  |  |
|  | CC | 108 (75.0) | 103 (71.5) | - |  |
|  | CT | 35 (24.3) | 40 (27.8) | 1.285 (0.692-2.387) |  |
|  | TT | 1 (0.7) | 1 (0.7) | 0.410 (0.012-13.939) | 0.306 |
|  | CT + TT vs CC | 36 (25.0) | 41 (28.5) | 1.228 (0.585-2.578) |  |
|  | Allele C | 87.15 | 85.71 |  |  |
|  | T | 12.85 | 14.29 |  |  |
|  | **rs2010963 (VEGF + 405 G/C)** |  |  |  |  |
|  | GG | 60 (41.7) | 55 (38.2) | - |  |
|  | GC | 71 (49.3) | 65 (45.1) | 0.980 (0.544-1.766) |  |
|  | CC | 13 (9.0) | 24 (16.7) | 1.389 (0.564-3.422) | 0.213 |
|  | GC + CC vs GG | 84 (58.3) | 89 (61.8) | 0.973 (0.470-2.017) |  |
|  | Allele G | 66.32 | 66.29 |  |  |
|  | C | 33.68 | 33.71 |  |  |
| **6p21** | **rs9295740 (vWF G/A)** |  |  |  |  |
|  | GG | 94 (65.3) | 84 (58.3) | - |  |
|  | GA | 42 (29.2) | 54 (37.5) | **1.978 (1.076 – 3.636)** |  |
|  | AA | 8 (5.6) | 6 (4.2) | 0.814 (0.229-2.895) | 0.263 |
|  | GA + AA vs GG | 50 (34.8) | 60 (41.7) | **1.742 (0.979-3.098)** |  |
|  | Allele G | 79.86 | 77.08 |  |  |
|  | A | 20.14 | 22.92 |  |  |
| **15q25** | **rs12914385 (CHRNA3 C/T)** |  |  |  |  |
|  | CC | 51 (35.4) | 39 (27.1) | - |  |
|  | CT | 69 (47.9) | 72 (50.0) | **1.835 (0.964 – 3.490)** |  |
|  | TT | 24 (16.7) | 33 (22.9) | 1.844 (0.824-4.126) | 0.935 |
|  | CT + TT vs CC | 93 (64.6) | 105 (72.9) | **1.837 (1.002-3.369)** |  |
|  | Allele C | 59.38 | 58.82 |  |  |
|  | T | 40.62 | 41.18 |  |  |
|  | **rs8034191 (LOC123688 T/C)** |  |  |  |  |
|  | TT | 53 (36.8) | 44 (30.6) | - |  |
|  | TC | 67 (46.5) | 71 (49.3) | **1.785 (0.947–3.362)** |  |
|  | CC | 24 (16.7) | 29 (20.1) | 1.424 (0.635-3.195) | 0.718 |
|  | TC + CC vs TT | 91 (63.2) | 100 (69.4) | **1.674 (0.925-3.032)** |  |
|  | Allele T | 60.07 | 61.39 |  |  |
|  | C | 39.93 | 38.61 |  |  |
|  | **rs931794 (AGPHD1 G/A)** |  |  |  |  |
|  | GG | 25 (17.4) | 26 (18.0) | - |  |
|  | GA | 65 (45.1) | 73 (50.7) | 1.460 (0.674 – 3.161) |  |
|  | AA | 54 (37.5) | 45 (31.3) | 0.839 (0.372 – 1.892) | 0.478 |
|  | AG + GG vs AA | 119 (82.6) | 118 (82.0) | 1.160 (0.563-2.389) |  |
|  | Allele A | 39.93 | 43.40 |  |  |
|  | G | 60.07 | 56.60 |  |  |

**Abbreviations:** NSCLC, non-small-cell lung cancer; VEGF, vascular endothelial growth factor; vWF, von Willebrand factor; CHRNA3, cholinergic nicotine receptor alpha3; VEGF 3'-UTR, VEGF 3’untranslated region; TERT, telomerase reverse transcriptase; CLPTM1L, cleft lip and palate transmembrane 1-like; AGPHD1, aminoglycoside phosphotransferase domain containing 1; HWE, Hardy-Weinberg equilibrium. In HWE column p > 0.05 stands for control group in HWE. **Bold** was used for highlight the almost statistical significance results and **bold + gray** for statistic significant results.
