# Supplementary material for: The Impact of Polymorphic Variations in the 5p15, 6p12, 6p21 and 15q25 Loci on the Risk and Prognosis of Portuguese Patients with Non-Small Cell Lung Cancer
Source: PLoS One. 2013 Sep 6;8(9):e72373. doi: 10.1371/journal.pone.0072373 (PMC3765163; doi:10.1371/journal.pone.0072373)
Supplement: Table S2 — Analysis of all 19 SNPs and patients outcome. (DOC) [file pone.0072373.s002.doc]

**Table 2 Supplementary** - genetic polymorphisms and non-small-cell lung cancer outcome (PFS, OS, RR for 1th line regimen).

| **Chromosome region** | **Genotype** | **PFS (months)*** | ***P* value** | **OS (months)*** | ***P* value** | **Positive RR (%)** | ***P* value** | **Positive RR (OR, 95% CI)Ɨ** |
| --- | --- | --- | --- | --- | --- | --- | --- | --- |
| **5p15** | **rs4635969 C/T (TERT)** |  |  |  |  |  |  |  |
|  | CC | 5 (3.38 – 6.61) |  | 9 (6.39 – 11.60) |  | 41.4 |  | - |
|  | CT | 5 (1.94 – 8.05) | 0.665 ^A^ | 15 (9.17 – 20.83) | 0.337 ^A^ | 41.9 | 0.66 **^C^** | 1.036 (0.427 – 2.517) |
|  | TT | 6 (0.001 – 16.73) |  | 11 (0.265 – 21.73) |  | 60.01 |  | 1.918 (0.290 – 12.672) |
|  | CT + TT vs CC | 5 (2.21 – 7.78) | 0.445 ^A^ | 13 (8.82 – 17.17) | 0.417 ^A^ | **44.4** | **0.006 ^C^** | 1.129 (0.486 – 2.623) |
|  | **rs31489 (CLPTM1L C/A)** |  |  |  |  |  |  |  |
|  | CC | 4 (1.89 – 6.10) |  | **6 (1.104 – 10.896)** |  | 33.3 |  | - |
|  | CA | 6 (4.16 – 7.83) | 0.588 ^A^ | **13 (6.33 – 19.66)** | **0.029 ^F^** | 43.5 | 0.316 ^C^ | 0.408 (0.122 – 1.360) |
|  | AA | 6 (1.42 – 10.57) |  | **13 (6.98 – 19.01)** |  | 55.6 |  | 0.625 (0.208 – 1.879) |
|  | CA + AA vs CC | 6 (4.42 – 7.57) | 0.449 ^A^ | **13 (7.89 – 18.1)** | **0.008 ^F^** | 46.9 | 0.216 ^C^ | 0.571 (0.231 – 1.416) |
|  | **rs401681 (CLPTM1L C/T)** |  |  |  |  |  |  |  |
|  | CC | **2 (0.945 – 3.05)** |  | **6 (3.02 – 8.97)** |  | 33.3 |  |  |
|  | CT | **5 (3.22 – 6.77)** | **0.021^D^** | **13 (8.61 – 17.38)** | **0.046** ^A^ | 43.1 | 0.442 ^C^ | 1.488 (0.538 – 4.116) |
|  | TT | **7 (0.001 – 14.04)** |  | **10 (5.10 – 14.89)** |  | 52.6 |  | 2.149 (0.620 – 7.455) |
|  | CT + TT vs CC | 6 (4.5 – 7.49) | 0.332 ^A^ | **11 (8.38 – 13.61)** | **0.021** ^A^ | 80 | 0.29 ^C^ | 1.644 (0.620 – 4.359) |
|  | **rs402710 (CLPTM1L C/T)** |  |  |  |  |  |  |  |
|  | CC | 5 (0.0001 – 11.23) |  | 10 (4.02 – 15.97) |  | 46.2 |  |  |
|  | CT | 6 (3.41 – 8.58) | 0.269 ^A^ | 13 (8.61 – 17.38) | 0.309 ^A^ | 48.8 | 0.397 ^C^ | 1.063 (0.303 – 3.721) |
|  | TT | 4 (1.62 – 6.37) |  | 6 (2.85 – 9.14) |  | 34.2 |  | 0.594 (0.164 – 2.148) |
|  | CT + TT vs CC | 5 (3.59 – 6.40) | 0.654 ^A^ | 10 (7.90 – 12.09) | 0.958 ^A^ | 42 | 0.777 ^C^ | 0.812 (0.248 – 2.654) |
| **6p12** | **rs3025035 (VEGF intron 7 C/T)** |  |  |  |  |  |  |  |
|  | CC | 5 (3.55 – 6.44) |  | 10 (7.28 – 12.71) |  | 35.5 |  | - |
|  | CT | 8 (3.88 – 12.11) | 0.282 ^A^ | 12 (4.35 – 19.65) | 0.472 ^A^ | **72.2** | **0.005 ^C^** | **4.90 (1.56 – 15.37)** |
|  | TT | - |  | - |  | - |  | - |
|  | **rs3025039 (VEGF +936 C/T)** |  |  |  |  |  |  |  |
|  | CC | 5 (3.57 – 6.42) |  | 11 (8.50 – 13.50) |  | 41.1% |  | - |
|  | CT | 7 (3.32 – 10.67) | 0.740 ^A^ | 10 (8.99 – 11.001) | 0.412 ^A^ | 47.6 | 0.080 ^C^ | 1.30 (0.489 – 3.457) |
|  | TT | - |  | 3 |  | - |  | - |
|  | **rs3025040 (VEGF 3'-UTR T/C)** |  |  |  |  |  |  |  |
|  | TT | - |  | 3 |  | - |  |  |
|  | TC | 7 (2 – 11.99) | 0.753 ^A^ | 10 (8.98 – 11.01) | 0.381 ^A^ | 47.6 | 0.594 ^C^ | 0.775 (0.291 – 2.060) |
|  | CC | 5 (3.64 – 6.35) |  | 11 (8.28 – 13.71) |  | 41.1 |  | - |
|  | TC + CC vs TT | 5 (3.6 – 6.4) | 0.460 ^A^ | 10 (8.122 – 11.87) | 0.203 ^A^ | 42.6 | - | - |
|  | **rs1005230 (VEGF -2489 C/T)** |  |  |  |  |  |  |  |
|  | CC | 5 (2.648 – 7.352) |  | 9 (3.772 – 14.228) |  | 35.7 |  | - |
|  | CT | 6 (4.688 – 7.312) | 0.424 ^A^ | 11 (8.059 – 13.941) | 0.992 ^A^ | 46 | 0.423 ^C^ | 1.529 (0.588 – 3.976) |
|  | TT | 3 (0.820 – 5.180) |  | 9 (2.097 – 15.903) |  | 43.8 |  | 1.304 (0.367 – 4.638) |
|  | CT +TT vs CC | 5 (3.140 – 6.590) | 0.985 ^A^ | 10 (8.287 – 11.713) | 0.696 ^A^ | 45.5 | 0.755 ^C^ | 1.472 (0.589 – 3.68) |
|  | **rs699947 (VEGFA - 2578 C/A)** |  |  |  |  |  |  |  |
|  | CC | 5 (2.64 – 7.35) |  | 9 (3.77 – 14.22) |  | 35.7 |  | - |
|  | CA | 6 (4.68 – 7.31) | 0.424 ^A^ | 11 (8.05 – 13.94) | 0.922 ^A^ | 46 | 0.674 ^C^ | 1.529 (0.588 – 3.976) |
|  | AA | 3 (0.82 – 5.18) |  | 9 (2.09 – 15.90) |  | 43.8 |  | 1.304 (0.367 – 4.638) |
|  | CA + AA vs CC | 5 (3.41 – 6.59) | 0.985 ^A^ | 10 (8.28 – 11.71) | 0.686 ^A^ | 45.5 | 0.382 ^C^ | 1.472 (0.589 – 3.68) |
|  | **rs833061 (VEGF – 460 C/T)** |  |  |  |  |  |  |  |
|  | CC | 3 (0.0001 – 6.76) |  | 9 (5.38 – 12.61) |  | **43.8** |  | - |
|  | CT | 5 (3.82 – 6.18) | 0.752 ^A^ | 11 (8.47 – 13.52) | 0.975 ^A^ | **42.9** | **0.036 ^C^** | 1.028 (0.331 – 3.196) |
|  | TT | 5 (0.939 – 9.061) |  | 10 (3.06 – 16.92) |  | **40.9** |  | 0.967 (0.258 – 3.626) |
|  | CT + TT vs CC | 5 (3.84 – 6.15) | 0.469 ^A^ | 10 (8.14 – 11.86) | 0.902 ^A^ | **42.3** | **0.011 ^C^** | 1.011 (0.336 – 3.039) |
|  | **rs833070 (VEGF intron 2 A/G)** |  |  |  |  |  |  |  |
|  | AA | 3 (0.82 – 5.18) |  | 9 (2.09 – 15.90) |  | 43.8 |  | - |
|  | AG | 6 (4.68 – 7.31) | 0.424 ^A^ | 11 (8.05 – 13.94) | 0.569 ^B^ | 46 | 0.788^C^ | 1.172 (0.372 – 3.697) |
|  | GG | 5 (2.64 – 7.35) |  | 9 (3.77 – 14.22) |  | 35.7 |  | 0.767 (0.216 – 2.728) |
|  | AG + GG vs AA | 5 (3.83 – 6.17) | 0.206 ^A^ | 10 (7.41 – 12.58) | 0.471^B^ | **42.6** | **0.011^C^** | 1.011 (0.336 – 3.039) |
|  | **rs3025010 (VEGF intron 5 C/T)** |  |  |  |  |  |  |  |
|  | CC | 4 (1.85 – 6.14) |  | **6 (3.16 – 8.83)** |  | 38.1 |  |  |
|  | CT | 6 (4.35 – 7.64) | 0.977 ^A^ | **13 (9.63 – 16.36)** | **0.047 ^D^** | 41.5 | 0.307 ^C^ | 1.163 (0.481 – 2.81) |
|  | TT | 6 (1.01 – 10.98) |  | **10 (0.0001 – 20.22)** |  | 63.6 |  | 2.733 (0.685 – 10.90) |
|  | CT + TT vs CC | 6 (4.39 – 7.60) | 0.883 ^A^ | **13 (8.22 – 11.17)** | **0.020 ^F^** | 46.2 | 0.530 ^C^ | 1.393 (0.979 – 1.053) |
|  | **rs3024994 (VEGF intron 2 T/C)** |  |  |  |  |  |  |  |
|  | TT | 6 (5.17 – 6.82) |  | 6 (1.26 – 10.73) |  | 18.2 |  | - |
|  | TC | 5 (3.21 – 6.78) | 0.475 ^A^ | 10 (7.33 – 12.66) | 0.394 ^A^ | 45.8 | **0.075** **^C^** | 3.604 (0.726 – 17.884) |
|  | CC | - |  | - |  | - |  | - |
|  | **rs25648 (VEGF – 7 C/T)** |  |  |  |  |  |  |  |
|  | CC | 5 (3.55 – 6.44) |  | 26 (0.0001 – 59.22) |  | 38 |  | - |
|  | CT | 5 (1.78 – 8.21) | 0.997 ^A^ | 45 (26.34 – 63.65) | 0.568 ^A^ | 54.5 | 0.087 ^C^ | 1.971 (0.747 – 5.20) |
|  | TT | 9 |  | 26 |  | 100 |  | - |
|  | CT + TT vs CC | 5 (0.714 – 9.286) | 0.994 ^A^ | 12 (8.76 – 15.23) | 0.569 ^A^ | 56.5 | 0.119 ^C^ |  |
|  | **rs2010963 (VEGF + 405 G/C)** |  |  |  |  |  |  |  |
|  | GG | 5 (3.1 – 6.89) |  | **9 (4.17 – 13.82)** |  | 47.1 |  | - |
|  | GC | 5 (3.12 – 6.87) | 0.463 ^A^ | **13 (8.70 – 17.29)** | **0.042 ^G^** | 43.2 | 0.570 ^C^ | 0.876 (0.354 – 2.164) |
|  | CC | 5 (1.67 – 8.32) |  | **3 (0.0001 – 8.88)** |  | 31.3 |  | 0.503 (0.143 – 1.771) |
|  | GC + CC vs GG | 5 (2.98 – 7.01) | 0.459 ^A^ | 10 (5.56 – 14.43) | 0.441 ^G^ | 40 | 0.506 ^C^ | 0.759 (0.324 – 1.779) |
| **6p21** | **rs9295740 (vWF G/A)** |  |  |  |  |  |  |  |
|  | GG | **4 (2.06 – 5.93)** |  | 9 (5.91 – 12.08) |  | **41.8** |  | - |
|  | GA | **6 (3.21 – 8.78)** | **0.074** ^A^ | 13 (8.4 – 17.59) | 0.107 ^A^ | **50** | **0.042 ^C^** | 1.385 (0.584 – 3.285) |
|  | AA | **4 (0.39 – 7.60)** |  | 11 (8.6 – 13.4) |  | 0.0001 |  | - |
|  | GA + AA vs GG | **6 (4.31 – 7.68)** | **0.034 ^A^** | **13 (9.38 – 16.61)** | **0.045 ^A^** | 43.6 | 0.864 ^C^ | 1.066 (0.464 – 2.452) |
| **15q25** | **rs12914385 (CHRNA3 C/T)** |  |  |  |  |  |  |  |
|  | CC | 6 (3.77 – 8.22) |  | 12 (2.46 – 21.53) |  | 34.6 |  |  |
|  | CT | 5 (3.35 – 6.64) | 0.550 ^A^ | 10 (6.54 – 13.45) | 0.761 ^A^ | 46.7 | 0.610 ^A^ | 1.801 (0.651 – 4.979) |
|  | TT | 6 (2.91 – 9.08) |  | 10 (6.48 – 13.51) |  | 43.5 |  | 1.511 (0.472 – 4.830) |
|  | CT + TT vs CC | 5 (3.28 – 6.71) | 0.318 ^A^ | 10 (8.11 – 11.88) | 0.697 ^A^ | 45.6 | 0.336 ^C^ | 1.693 (0.653 – 4.388) |
|  | **rs8034191 (LOC123688 T/C)** |  |  |  |  |  |  |  |
|  | TT | 6 (4.30 – 7.69) |  | 12 (3.96 – 20.03) |  | 35.7 |  |  |
|  | TC | 4 (2.59 – 5.40) | 0.428 ^A^ | 10 (5.19 – 14.81) | 0.840 ^A^ | 45.5 | 0.683 ^C^ | 1.633 (0.604 – 4.411) |
|  | CC | 6 (4.79 – 7.20) |  | 10 (8.24 – 11.75) |  | 45.5 |  | 1.568 (0.497 – 4.948) |
|  | TC + CC vs TT | 5 (3.56 – 6.43) | 0.200 ^A^ | 10 (7.80 – 12.19) | 0.872 ^A^ | 45.5 | 0.382 ^C^ | 1.610 (0.637 – 4.069) |
|  | **rs931794 (AGPHD1 G/A)** |  |  |  |  |  |  |  |
|  | GG | 6 (4.86 – 7.13) |  | 10 (8 – 11.99) |  | 40 |  | - |
|  | GA | 4 (2.67 – 5.32) | 0.318^A^ | 10 (5.71 – 14.28) | 0.676 ^A^ | 44.4 | 0.934 ^C^ | 1.240 (0.422 – 3.647) |
|  | AA | 6 (3.95 – 8.05) |  | 15 (7.74 – 22.25) |  | 41.4 |  | 1.032 (0.322 – 3.312) |
|  | GA + AA vs GG | 5 (3.38 – 6.61) | 0.605 ^A^ | 10 (6.62 – 13.37) | 0.752 ^A^ | 43.2 | 0.795 ^C^ | 1.154 (0.420 – 3.167) |

**Abbreviations**: * median; PFS, progression-free-survival; OS, overall survival; RR, response rate for 1th line therapy (complete, partial, stable); 95% CI, confidential interval 95%. ^A^ Log rank test; ^B^ Breslow test; ^C^ Chi square test; ^D^ Breslow test (only non-squamous cell tumors); ^E^ Log rank test (only stages IIIB and IV); ^F^ Log rank test (only non-squamous cell tumors); ^G^ Breslow test (only stages IIIB and IV). **Bold** was used for highlight almost statistical significance results and **bold + gray** for statistic significant results. ; **Ɨ** adjusted for age**.**
